# Adoption and uptake of the lateral flow urine LAM test in countries with high tuberculosis and HIV/AIDS burden: current landscape and barriers

**DOI:** 10.12688/gatesopenres.13112.2

**Published:** 2020-04-07

**Authors:** Diane N. Singhroy, Emily MacLean, Mikashmi Kohli, Erica Lessem, David Branigan, Kathleen England, Khairunisa Suleiman, Paul K. Drain, Morten Ruhwald, Samuel Schumacher, Claudia M. Denkinger, Brenda Waning, Wayne Van Gemert, Madhukar Pai

**Affiliations:** 1 McGill International TB Centre, McGill University, Montreal, QC, H4B1X5, Canada; 2 Department of Epidemiology, Biostatistics, and Occupational Health, McGill University, Montreal, QC, Canada; 3 Department of Tuberculosis, Treatment Action Group, New York, NY, USA; 4 Independent Consultant, Los Angeles, CA, USA; 5 Independent Consultant, Istanbul, Turkey; 6 Departments of Global Health, Medicine, and Epidemiology, University of Washington, Seattle, WA, USA; 7 Department of Tuberculosis, Foundation for Innovative New Diagnostics (FIND), Geneva, Switzerland; 8 Division of Tropical Medicine, Center of Infectious Diseases, University of Heidelberg, Heidelberg, Germany; 9 Stop TB Partnership, Global Drug Facility, Geneva, Geneva, Switzerland

**Keywords:** tuberculosis, lipoarabinomannan, LAM, rapid testing, urine, policy, barriers

## Abstract

**Background:** Since 2015, the World Health Organization (WHO) has recommended a commercially available lateral-flow urine LAM test (Alere-LAM) to assist in the diagnosis of tuberculosis (TB) in severely ill people living with HIV (PLHIV). The test can rapidly detect TB in severely ill PLHIV and can identify PLHIV most at-risk of death, leading to mortality reductions. However, its uptake in countries with high burdens of TB and HIV has been slow. To assess the current use landscape and identify barriers to the adoption of Alere-LAM, we conducted a questionnaire-based study in 31 high TB and HIV/AIDS burden countries.

**Methods**: Between November 2018 and December 2019, we collected responses to a semi-structured questionnaire that had been emailed to staff and affiliates of National TB Programs or HIV/AIDS Programs, Ministries of Health, and TB or HIV institutes of 31 high TB/HIV burden countries. Questions concerned country policies, adoption, and current use of Alere-LAM testing, as well as testing algorithms and barriers preventing Alere-LAM uptake.

**Results:** We received questionnaire responses from 24 out of 31 (77%) high TB/HIV burden countries. Of these 24 countries, 11 (46%) had adopted Alere-LAM policies, with only five (21%) countries currently using Alere-LAM testing. Testing algorithms were generally aligned with WHO recommendations. Fifteen countries (63%) said they were planning to implement Alere-LAM testing in the near future. The most commonly cited constraint to adoption and implementation was budget limitations. Additional barriers to Alere-LAM implementation included lack of country-specific data and piloting, administrative hurdles such as regulatory agency approval, lack of coordination between National TB and HIV programs, and small perceived patient population.

**Conclusion:** Responses to our questionnaire demonstrate the persistent gap between country-level policy and real-world use of Alere-LAM, as well as specific barriers that must be addressed to scale-up testing in PLHIV.

## Introduction

Tuberculosis (TB) is the leading cause of both hospitalization and death among people living with HIV/AIDS (PLHIV) ^1^. PLHIV are 26 times more susceptible to TB infection than those who are seronegative and in 2018, the World Health Organization (WHO) estimated 860 000 PLHIV had developed active TB disease ^2^. Further, over a third of deaths in PLHIV are attributed to TB co-infection ^3^. Yet, according to a systematic review of post-mortem studies in resource-limited settings, TB was undiagnosed in PLHIV until the time of death in 46% of cases ^4^.

The high rate of undiagnosed TB is partially a function of the available TB diagnostic tests that detect *Mycobacteria tuberculosis* ( *M. tuberculosis*) bacilli in respiratory samples, such as sputum. For many PLHIV, producing sputum is difficult and bacterial burden is low, resulting in diagnostic test sensitivities that are lower than in HIV-uninfected individuals ^5^. As well, PLHIV often present with extrapulmonary TB, for which sputum is not the correct sample.

Next generation assays such as Xpert MTB/RIF Ultra, a WHO-recommended PCR-based rapid TB test, offer improved diagnostic accuracy over conventional smear microscopy ^6^, but are often not available where patients first seek care due to infrastructure needs and relatively high cost ^7, 8^. Until accurate diagnostic tests for PLHIV become widely accessible, many individuals with TB will continue to be missed.

Lipoarabinomannan (LAM) is a component of the *M. tuberculosis* cell wall that can be detected in urine ^9, 10^. The first commercially available TB test based on urinary LAM detection is the Determine™ TB LAM Antigen assay (Alere-LAM), originally manufactured by Alere Inc. (now produced by Abbott). Available since 2013, this lateral flow LAM strip test requires 60 μl of urine and provides results in approximately 30 minutes ^11^. Besides a micropipette, no equipment is required, allowing its placement at the point-of-care, with a low cost of about 3.50 USD per test. Because the assay detects LAM in the urine, it is more likely than other sputum-based TB tests to diagnose PLHIV with extrapulmonary or advanced disease since urinary LAM concentrations are higher at lower CD4+ cell counts ^12^.

In 2015, the WHO published guidelines recommending the Alere-LAM test be used to assist TB diagnosis in hospitalized HIV-positive patients with TB symptoms and CD4+ cell counts less than or equal to (≤) 100 cells/µl, or who are seriously ill regardless of CD4+ cell count. Based on generalization of data from inpatients, these recommendations could be extended to adult HIV-positive ambulatory (outpatient) cases and, with discretion, to HIV-positive children while acknowledging lower specificity in this group ^13^.

In 2019, the Guidelines were updated to indicate the increased strength of the recommendation for Alere-LAM in hospitalized HIV-positive patients with CD4+ counts ≤ 200 cells/µl, or who are seriously ill regardless of CD4+ cell count; in this setting, the test is now more clearly recommended for children and adolescents. With respect to ambulatory settings, Alere-LAM testing is conditionally recommended for severely ill HIV-positive outpatients of all ages with CD4+ counts ≤ 100 cells/µl regardless of signs and symptoms, or any outpatient with TB symptoms. Use of Alere-LAM is discouraged without assessment of patient signs and symptoms and without knowing CD4+ cell count ^14^. The Cochrane systematic review and meta-analysis commissioned for the update found the pooled sensitivity of Alere-LAM was 42% (95% credible interval [CrI]: 31–55%) and pooled specificity was 91% (95%CrI: 85–95%) from eight studies and 3449 participants. In hospitalized in-patients, pooled sensitivity was 52% (95%CrI: 40–64%) versus 29% (95%CrI: 17–47%) among outpatients ^15^.

Despite the existence of these Guidelines, a 2018 Médecins Sans Frontières (MSF) report ^16^ as well as a Treatment Action Group (TAG) Activist Guide to TB LAM ^17^ found that very few high burden countries had adopted Alere-LAM in their national policies or articulated plans to scale up use of the test. We conducted a questionnaire-based study in 31 high TB and HIV/AIDS burden countries to assess the current landscape and identify barriers to the adoption of Alere-LAM.

## Methods

A semi-structured questionnaire was emailed to staff and affiliates of National TB Programs (NTPs) or HIV/AIDS Programs (NAPs), Ministries of Health, and TB or HIV institutes of 31 high TB/HIV burden countries, primarily identified from prior country surveys. The questionnaire is available as *Extended data* ^18^. Countries were chosen based on 2015 WHO classification ^19^. Questionnaire responses were collected between November 2018 and December 2019 and participants were contacted an average of three times for questionnaire responses. In cases of unclear comments, respondents were contacted for follow-up. By filling in the questionnaire, respondents consented for the provided data (de-identified) to be used for research purposes only. As the questionnaire only collected country level data, no ethical approval was required.

The questionnaire included ten questions and space for respondents to provide additional comments. Questions pertained to country adoption of Alere-LAM policies; current use of Alere-LAM; location of current testing; testing algorithms and use-cases; possible future test implementation and use; and barriers preventing Alere-LAM uptake and routine use. Respondents had the option to choose from a list of 17 suggested obstacles or could describe other constraints.

## Results

Out of the 31 high TB burden countries that were contacted, we received questionnaire responses from 33 individuals representing 24 countries (77%). The majority of respondents were affiliated with country NTPs (24/33, 73%), with five respondents from NAPs (5/33, 15%), two respondents with joint affiliations (2/33, 6%), and one individual from neither Program (1/33, 3%). All questionnaire responses are available as *Underlying data* ^18^.

**Figure 1.**
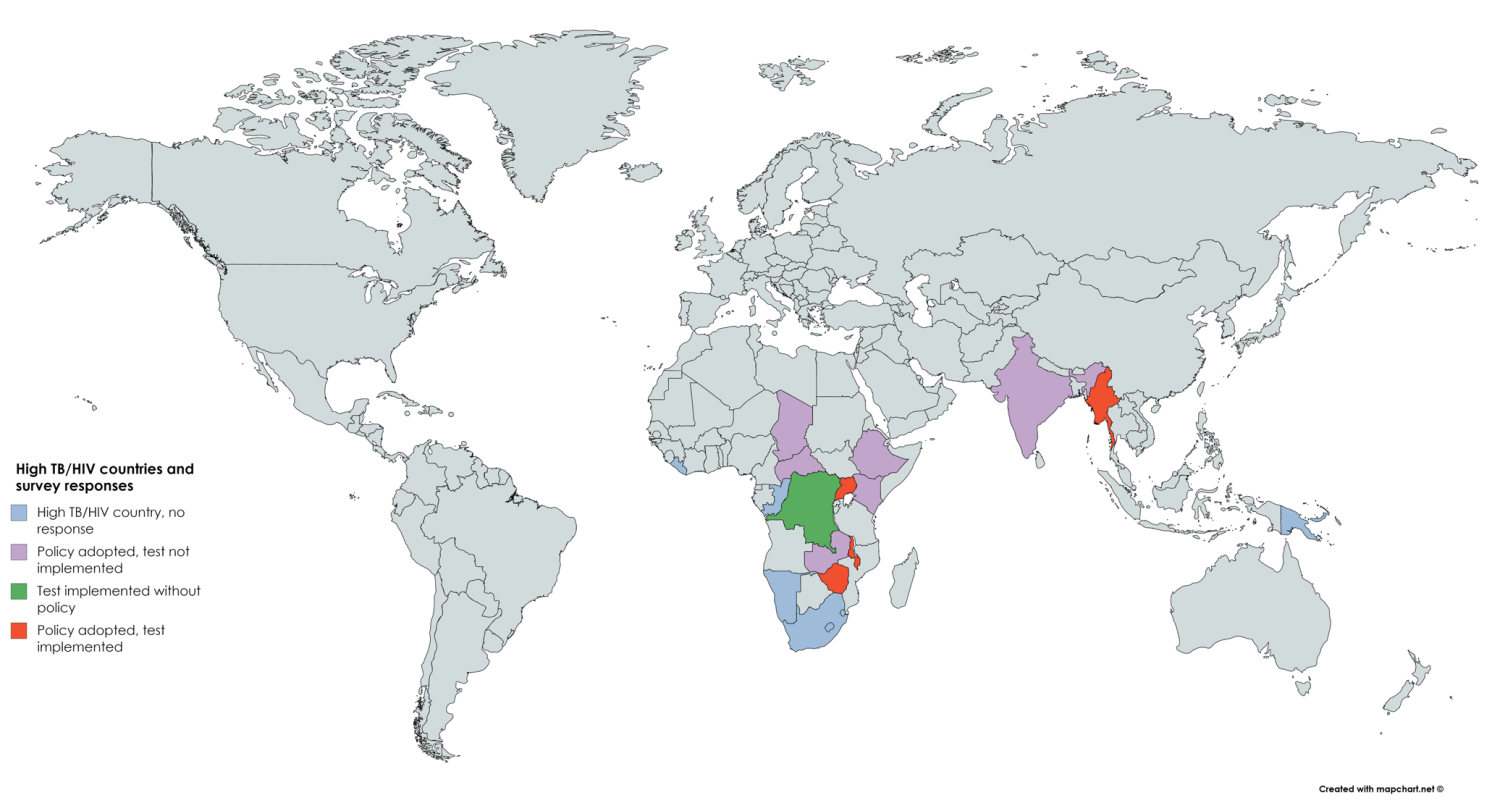
High TB/HIV burden countries contacted for survey. Status of Alere-LAM policy adoption and test implementation are as indicated by colour. Blue indicates countries that were contacted but did not respond to the survey. Purple indicated countries with an Alere-LAM policy but that have not implemented the test. Green indicates that Alere-LAM testing has been implemented without a policy. Red indicates that the country has an Alere-LAM policy and testing has been implemented.

Of the 24 countries, 11 (46%) had Alere-LAM policies at the time of questionnaire response. Of these 11, six countries had Alere-LAM policies in both NTPs and NAPs (55%), three countries had policies within the NTP only (27%), and two countries reported an Alere-LAM policy in their NAP only (18%) ( Table 1). But inclusion in policy does not mean actual usage. Notably, only four countries that had adopted an Alere-LAM policy within the NTP and/or NAP had implemented testing. One country, the Democratic Republic of Congo (DRC) was utilizing Alere-LAM testing without a policy in either Program ( Figure 1). When asked if they were planning to use Alere-LAM in the near future, 15 countries (15/24, 63%) responded yes ( Table 1). Altogether, 20 of 24 countries (83%) were currently using or were planning to deploy Alere-LAM testing.

**Table 1.** Stages of implementation of Alere-LAM urine test by country.

Country	Does the country have policies that include Alere-LAM? (year of introduction) ^a^	Which National Program includes Alere-LAM? (TB, HIV, both) ^b^	Is Alere- LAM currently in use? ^c^	Is Alere-LAM use planned for the near future? (prospective year) ^d^
**Angola**	No	-	No	Yes (2019)
**Botswana**	Unclear ^e^ (2018)	TB	No	Yes (2019)
**Brazil**	No	-	No	Yes
**Cameroon**	-	-	No	Yes
**CAR**	Yes (2016)	Both	No	Yes (2019)
**Chad**	Yes (2018)	TB	No	Yes
**China**	No	-	No	No
**DRC**	No	-	Yes	Yes
**Ethiopia**	Yes (2017)	Both	No	Yes
**Ghana**	No	-	No	Do not know
**Guinea-Bissau**	No	-	No	No
**India**	Yes ^f^	HIV	No	Yes (2020)
**Indonesia**	No	-	No	Yes
**Kenya**	Yes (2019)	Both	No	Yes
**Malawi**	Yes (2017)	Both	Yes	-
**Mozambique**	No	-	-	-
**Myanmar**	Yes (2017)	HIV	Yes	Yes
**Nigeria**	No	-	-	Yes (2019)
**Philippines**	No	-	No	Yes (2021)
**Tanzania**	No	-	No	No
**Thailand**	No	-	No	No
**Uganda**	Yes (2017)	Both	Yes	-
**Zambia**	Yes (2018)	TB	No	Yes
**Zimbabwe**	Yes (2017)	Both	Yes	-
**Number answering** **“yes”**	**10**	**NA**	**5**	**15**

Summary of answers given by respondents regarding Alere-LAM test adoption stage. A dash indicates no response. CAR = Central African Republic. DRC = Democratic Republic of Congo. ^a^Years that Alere-LAM test policies were introduced into either the TB or HIV National Programs are listed in brackets as reported by the respondents. ^b^The questionnaire was limited to Alere-LAM policy inclusion in TB and HIV National Programs. ^c^Actual use of the Alere-LAM urine tests is independent of existence of a national policy. ^d^Prospective year was included only if reported by respondents. ^e^Respondents gave conflicting responses. ^f^Test is not yet introduced.

Respondents from ten of the eleven countries with Alere-LAM policies provided details regarding their testing algorithms with respect to the then-current 2015 Guidelines ( Table 2) ^13^. As expected, policies in eight of ten countries (80%) followed these guidelines. That is, eight of ten countries were using the Alere-LAM test in hospitalized PLHIV with CD4+ counts ≤100 cells/µl, and seven of ten (70%) countries were using the test in seriously ill PLHIV inpatients ( Table 2). Notably, in Mozambique, the NAP is discontinuing CD4+ cell testing, so CD4+ cell count was not part of their Alere-LAM testing algorithm. Six countries (6/10, 60%) were using the test in children. Four countries were using the Alere-LAM test in outpatient scenarios ^14^.

**Table 2.** Alere-LAM testing algorithms and its location of use.

	Botswana	C. African Rep.	Chad	DRC	Kenya	Malawi	Mozambique	Myanmar	Uganda	Zimbabwe	TOTAL (10)
Current algorithm for Alere-LAM testing											
To assist in the diagnosis of TB in **adult** HIV positive **in-patients** with a CD4 count ≤ 100 cells/µL	X	X	X	X	X	X			X	X	8
To assist in the diagnosis of TB in **children** HIV positive **in-patients** with a CD4 count ≤ 100 cells/µL			X		X	X			X		4
To assist in the diagnosis of TB in **adult** HIV positive **out-patients** (ambulatory) with a CD4 count ≤ 100 cells/µL			X						X	X	3
To assist in the diagnosis of TB in **children** HIV positive **out-patients** (ambulatory) with a CD4 count ≤ 100 cells/µL			X						X		2
To assist in the diagnosis of TB in **adult** HIV positive **in-patients** who are seriously ill regardless of CD4 count or with unknown CD4	X				X	X	X	X	X	X	7
To assist in the diagnosis of TB in **children** HIV positive **in-patients** who are seriously ill regardless of CD4 count or with unknown CD4					X	X	X	X	X		5
To assist in the diagnosis of TB in **adult** HIV positive **out-patients** (ambulatory) who are seriously ill regardless of CD4count or with unknown CD4							X		X	X	3
To assist in the diagnosis of TB in **children** HIV positive **out-patients** (ambulatory) who are seriously ill regardless of CD4count or with unknown CD4							X		X		2
To assist in the diagnosis of TB in **adult** HIV positive **in-patients** regardless of CD4 count or with unknown CD4	X				X				X		3
To assist in the diagnosis of TB in **children** HIV positive **in-patients** regardless of CD4 count or with unknown CD4					X				X		2
Where is the Alere-LAM test being used?											
All hospitals			X			X			X	X	4
Limited number of hospitals							X	X			2
All Antiretroviral Therapy (ART) centers			X						X		2
Limited number of ART centers				X							1
Other (Pilot in 12 high volume facilities, to start)					X						1

Algorithm is based on WHO 2015 Lateral Flow LAM guidelines. Although India has an Alere-LAM policy, algorithm details were not provided.

Availability of testing varied substantially across countries. Eight countries reported the setting where Alere-LAM testing was available ( Table 2). In Chad and Uganda, Alere-LAM was most widely available, as testing is offered in all hospitals and antiretroviral treatment (ART) centers. Malawi and Zimbabwe provide Alere-LAM testing at all hospitals. Myanmar and the DRC provide Alere-LAM testing at a limited number of hospitals or ART centers. An Alere-LAM testing pilot program was limited to 12 hospitals in Kenya. Mozambique reported that they had been using the Alere-LAM test within a research context only (i.e., not a pilot). Botswana, Central African Republic, and India have Alere-LAM policies, but the test is not yet in use and eventual test use locations were not reported.

**Figure 2.**
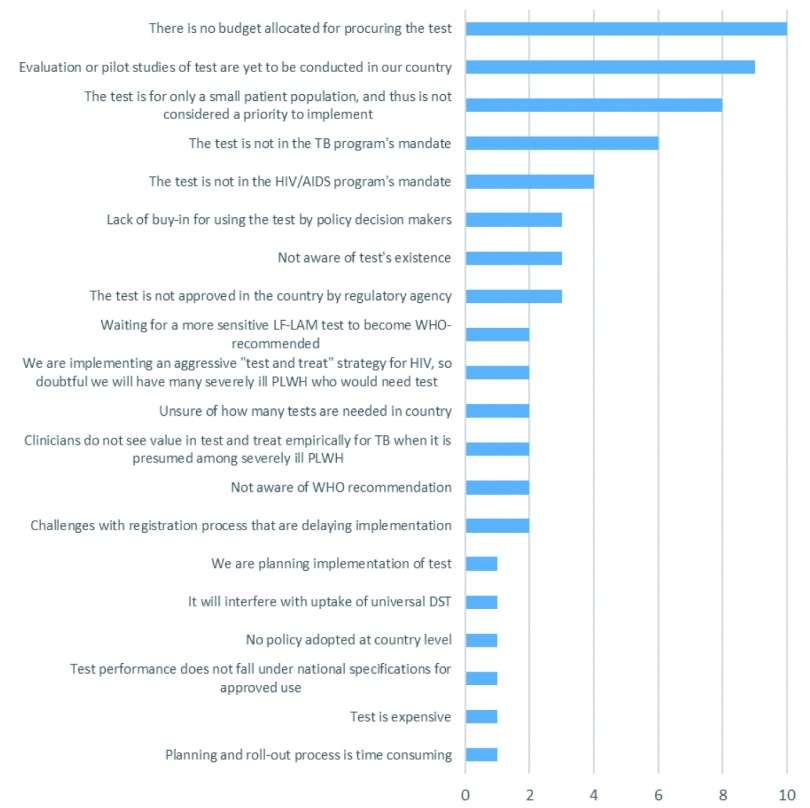
Barriers to Alere-LAM urine test use. Questionnaire included a set list of barriers and allowed respondents to report other barriers not included. Unique barriers per country are reported here. For example, if two surveys from one country listed the same barrier, it was only included once.

Almost all countries (21/24, 88%) identified barriers preventing implementation of Alere-LAM ( Figure 2). Selected comments from questionnaires are quoted in Box 1. Budget limitations were the most reported barrier (10/21, 48%). Some countries had indicated that they were currently consulting with financial partners to support uptake or that they hope to use Alere-LAM as soon as funding becomes available in their country ( Figure 2). The second most reported barrier was that pilot studies or evaluations within their country needed to be conducted. In that context, some countries that reported this explanation already had ongoing studies, but were waiting on results to inform their guidelines, determine the best use and placement in national diagnostic algorithms, and assess accuracy along with various operational issues.

**Box 1. Select comments from country surveys**

**Comment**

    •   “[We] are ready to use the test. However, the NTP with the NAP are planning to carry out a pilot study not only to assess the accuracy of the test but also the operational issues.”

    •   “This test is crucial for [us] to improve diagnostic capacity for national TB program. I suggest, piloting of this test is important to show added value for TB diagnosis due to [Alere-LAM].”

    •   “The test was tried in a small PhD study [here]. It proved very useful but has not been considered for use because of the existing tests in use (Smear microscopy, GeneXpert, LPA and liquid culture). It is also considered to be useful in a small population of patients (severely ill HIV/TB co-infected persons).”

    •   “The test is currently under verification by a team from TB and HIV programs. The report will be used for implementation.”

    •   “Overall, we think that this rapid test that uses urine (a much easier sample to obtain), is a breakthrough with potential to help to find the missing cases.”

    •   “We believe that discontinuing CD4 by the HIV Program may negatively impact the decision whether or not to use the test and increase the risk of using the test in people with elevated CD4, producing false negatives.”

    •   “In 2018, it was planned to procured to start the implementation in HIV specialist hospitals and planned to roll out to all ART centers. However, when the international procurement through UNOPS was proceeded, the supplier could not import the product without country FDA’s approval letter.”

    •   “In the NTP perspective, in an environment like ours where there is a test and treat policy and therefore very few anticipated HIV patients would be severely ill, the usefulness of this test needs to be well demonstrated specifically to the HIV program where most of these patients are found. The NTP program's experience with rolling out IPT program was very challenged because of introducing the program via the NTP with no ownership of the HIV program.”

    •   “The policy guidelines which introduced TB-LAM were developed in 2017 but actual implementation started in 2018 because of delays in receiving the tests. The [then] current guidelines from WHO do not seem to explicitly recommend the use of the test in children and our guidelines have taken the same bias.”

Some countries had indicated that implementing Alere-LAM was not a priority because of the relatively small size of the population affected: this was the third most common barrier. In certain cases, this belief prevailed because NTPs/NADs did not expect a high number of seriously ill PLHIV cases due to aggressive existing “test and treat” programs. Other barriers identified were more administrative in nature. For example, the purview of Alere-LAM testing was reportedly not within the NAP or NTP, Alere-LAM was not approved by country-relevant regulatory agencies, challenges persist with registering the test in the country, and estimates of the number of required tests needed for country-wide implementation were reported as uncertain.

## Discussion

Despite the positive WHO recommendations and evidence demonstrating that Alere-LAM testing reduces mortality in the intended use population ^20^, our analysis is consistent with previous reports ^16, 17^ in finding that Alere-LAM use remains low among high TB/HIV burden countries. As we observed directly from the questionnaire responses, existence of policy for a test does not guarantee routine use, as there are numerous barriers to overcome before country-level implementation of a test can begin.

While policies recommending the use of Alere-LAM have been adopted by eleven (11/24, 46%) countries at the time of data collection, the test was routinely used in only four of these countries, plus the DRC (which is using Alere-LAM without a policy) ( Figure 1). Despite the severity of the HIV-TB co-epidemic, the generally siloed nature of HIV/AIDS and TB care is also observed in the context of Alere-LAM testing: six countries had adopted Alere-LAM policies in both NTPs and NAPs, but in two and in three countries, only the NAP and NTP, respectively, had an Alere-LAM policy. This distinct separation was further reflected in the reported barriers to Alere-LAM uptake: four countries stated that the Alere-LAM test is not within the scope of the NAP and six responded that it is not within the NTP’s mandate.

For PLHIV who are coinfected with TB, this lack of collaboration manifests in particularly low rates of TB testing, diagnosis, and eventual TB treatment completion ^21^. A systemic review and meta-analysis of post-mortem studies found that approximately 40% of deaths in PLHIV were attributable to TB, but half of these TB cases were undiagnosed in the person’s lifetime ^4^. By employing Alere-LAM, many of these deaths could have been prevented. In a 2016 systematic review and meta-analysis, the risk of mortality was 2.3 times higher in PLHIV who tested LAM-positive than in PLHIV who tested negative, so this test could be used as a life-saving prognostic tool ^22^. Indeed, it has been shown that urine is more feasible to collect and testing can occur within 24 hours of hospital admission ^23^, and that routine Alere-LAM testing in hospitalized PLHIV led to a 4% (95%CI: 7–9%) absolute risk reduction in death, as clinicians could identify patients who needed rapid anti-TB treatment initiation ^24^.

Issues surrounding Alere-LAM jurisdiction were not the only barriers to the test’s implementation. In-country test piloting was commonly cited, and while such evaluation is important for understanding logistical and country-specific implementation issues, they can significantly delay the introduction of new tests where they are most needed. Additionally, the belief that only a small volume of people would require Alere-LAM testing, due to the existence of “test and treat” programs and ART scale-up, prevails. Unfortunately, this claim is not substantiated by the data. In a retrospective cohort study of over 8 million CD4+ cell count test results in South Africa, the proportion of people presenting to care with advanced HIV disease was 33%, relatively unchanged since 2011, and those presenting with very advanced disease was 17% ^25^.

Budget limitations were the most commonly cited barrier (10/21, 48%). This is despite the low end-user cost per test cost of Alere-LAM, approximately 3.50 USD via Global Drug Facility ^16, 26^, well below the WHO Target Product Profile optimal cost for a rapid non-sputum-based biomarker test for TB detection ^27^. The lack of budget allocation may be related to the absence of targeted donor programs or concessional pricing negotiations around Alere-LAM’s launch, such as what was observed with Unitaid-funded access grants ^28^ or donor-orchestrated buydowns ^29^ in the case of Xpert MTB/RIF. It may also be a reflection of the WHO’s previously conditional (now strong) recommendations that were limited to a relatively small subgroup of PLHIV. The conditionality of the recommendations was perceived as a lack of confidence in the data, which likely played out at the country-level as a lack of enthusiasm for Alere-LAM, translating into a lack of political will, and thus funds, to improve test implementation and accessibility. Domestic funding for health systems must be grown; in conjunction with strategies that previously led to successful procurement of Xpert MTB/RIF, like those mentioned above, and novel solutions, such as reducing price mark-ups on in-country suppliers or pooling procurements from different regions, this should allow for increased availability of Alere-LAM. Hopefully, the now strong recommendation for Alere-LAM use in an expanded population will result in increased funding and implementation of testing.

Country testing policies for Alere-LAM, when existent, were generally (8/10, 80%) aligned with WHO guidelines, i.e. to assist in the diagnosis of TB in severely ill PLHIV or PLHIV with CD4+ counts ≤100 cells/µl. However, as WHO’s Consolidated ART Guidelines now recommend viral load testing for ART response monitoring instead of CD4+ count ^30^, there might be some uncertainty with regard to whom the test should be offered and whether the test can be used at decentralized facilities without clinical laboratories. The 2019 WHO Essential Diagnostics List includes Alere-LAM, but only at facilities with clinical laboratories ^31^. Although the updated LAM Guidelines depends significantly less on CD4+ cell counts than the 2015 recommendations, this ambiguity will need to be clarified by WHO, particularly for countries that are planning to implement Alere-LAM but currently have no policy or testing algorithm in place. It should be noted that the 2019 LAM Guidelines do strongly recommend the use of Alere-LAM in all severely ill PLHIV inpatients with TB symptoms, regardless of CD4+ cell count ^14^, which is now aligned with the ART Guidelines’ strong recommendation to initiate ART in all PLHIV regardless of clinical stage or CD4+ cell count ^30^.

Qualitative research published alongside the latest WHO Alere-LAM policy reported that among primary care health workers in high HIV/TB burden settings, confidence in Alere-LAM may be rather low, given its overall low sensitivity. As well, Alere-LAM is not a stand-alone diagnostic test, and TB rule-out requires confirmation by other methods, which, besides being difficult to obtain from severely ill PLHIV, may be difficult to explain to patients, lowering its acceptability. Finally, in order to have confidence in a positive Alere-LAM result, existing clinical suspicion on the part of clinicians remains necessary ^32^.

The primary limitation of this study is that although we surveyed the 31 highest TB/HIV burden countries, we did not attain a 100% response rate. However, it was relatively high (24/31, 77%), with most questionnaire responses coming from NTP employees. Lack of adequate responses from NAPs, however, could mean that we under-estimated Alere-LAM uptake. Also, we did not account for Alere-LAM use via PEPFAR, a major funder of ART scale-up in many high HIV prevalent settings.

Despite limitations, our study identified limited uptake of Alere-LAM among high TB/HIV burden countries. The barriers identified in our questionnaire, along with advocacy reports by groups such as TAG and MSF, can help policy makers, country governments, and donors build better strategies for Alere-LAM implementation and scale-up. These questionnaire results should help guide efforts to develop higher-sensitivity urine LAM tests ^33– 35^ and rapidly roll them out, as country adoption of improved products must anticipate challenges from the field.

## Data availability

### Underlying data

Open Science Framework: LAM country survey, https://doi.org/10.17605/OSF.IO/6W8VX ^18^.

This project contains the following extended data:

- Questionnaire responses from all completed questionnaires received.

### Extended data

Open Science Framework: LAM country survey, https://doi.org/10.17605/OSF.IO/6W8VX ^18^.

This project contains the following extended data:

- Questionnaire

Data are available under the terms of the Creative Commons Zero "No rights reserved" data waiver (CC0 1.0 Public domain dedication).
