# Supplementary material for: Adoption and uptake of the lateral flow urine LAM test in countries with high tuberculosis and HIV/AIDS burden: current landscape and barriers
Source: Gates Open Res. 2020 Apr 7;4:24. Originally published 2020 Feb 13. [Version 2] doi: 10.12688/gatesopenres.13112.2 (PMC7059561; doi:10.12688/gatesopenres.13112.2)
Supplement: Peer review history

# Peer review: Reviewer response for version 1

**DOI:** 10.21956/gatesopenres.14286.r28586

**Published:** 2020-03-02

**Authors:** Zibusiso Ndlovu

**Affiliations:** 1 Southern Africa Medical Unit, Medecins Sans Frontières, Cape Town, South Africa

This manuscript by Diane *et al.* on "Adoption and uptake of the lateral flow urine LAM test in countries with high tuberculosis and HIV/AIDS burden: current landscape and barriers" is a well written article and quite relevant. Poor utilization of diagnostics is now a big concern more than availability of treatments (in many cases).

 One of the identified major causes of poor adoption of urine TB LAM is mentioned as lack of funding. In the Discussion, the authors ought to have further mentioned the need for countries to grow domestic funding, especially for not so expensive tests like urine TB LAM. Furthermore, there is a lot of innovations that could be mentioned including: pooled regional procurements (as we know volumes based procurements can result in price reductions), waiver of taxes on global health products within countries, reductions on price mark-ups from in-country suppliers etc.

 This paper ought to give quite clear and pragmatic recommendations on key barriers to adoption.

I confirm that I have read this submission and believe that I have an appropriate level of expertise to confirm that it is of an acceptable scientific standard.

# Response

**Published:** 2020-04-01

**Authors:** Madhukar Pai

**Affiliations:** McGill University, Canada

**Thank you to the reviewers for your time and detail in evaluating our paper. Please find our comments addressing your comments directly below, in bold.**

This manuscript by Diane et al. on "Adoption and uptake of the lateral flow urine LAM test in countries with high tuberculosis and HIV/AIDS burden: current landscape and barriers" is a well written article and quite relevant. Poor utilization of diagnostics is now a big concern more than availability of treatments (in many cases).

One of the identified major causes of poor adoption of urine TB LAM is mentioned as lack of funding. In the Discussion, the authors ought to have further mentioned the need for countries to grow domestic funding, especially for not so expensive tests like urine TB LAM. Furthermore, there is a lot of innovations that could be mentioned including: pooled regional procurements (as we know volumes based procurements can result in price reductions), waiver of taxes on global health products within countries, reductions on price mark-ups from in-country suppliers etc.

**This suggestion has been incorporated into the Discussion (paragraph 6).**

This paper ought to give quite clear and pragmatic recommendations on key barriers to adoption.

**This suggestion has been incorporated into the Discussion (paragraph 6).**

# Peer review: Reviewer response for version 1

**DOI:** 10.21956/gatesopenres.14286.r28585

**Published:** 2020-03-02

**Authors:** María Elvira Balcells

**Affiliations:** 1 Department of Adult Infectious Diseases, School of Medicine, Pontifical Catholic University of Chile (UC), Santiago, Chile

This is an interesting study. It aims at reaching better knowledge of the local landscape and barriers for Alere-LAM tuberculosis test implementation in high TB and HIV burden countries.

 The study is relevant because the current version of this test has the disadvantage of having a suboptimal sensitivity for TB diagnosis, which can look unattractive to health care organizations from a public health perspective. However, the test can still be very valuable from both patients and clinicians perspectives: it is simple to perform, low-cost, and it can rapidly confirm severe forms of TB in up to 42% of cases with advanced HIV infection, decreasing these patients’ risk of death.

 On the whole, I was very well impressed with the paper.

 I only have a few minor points to mention: 

1. As one of the study objectives, the authors meant to assess the current landscape of Alere-LAM use in high TB and HIV burden countries. It is striking that 7 out of 11 respondents reported having included the test in their national policies, but not having implemented it yet. I wonder whether or not the authors verified local policies and algorithms for Alere-LAM testing for each of these countries. If not, the discussion should mention if a response bias may have partially accounted for this finding, which could have been particularly relevant when only one respondent participated per country.
2. It is an interesting finding of this study that the need for “evaluation or local pilots studies” was the second most reported barrier for Alere-LAM urine test use. This, which is a common practice in many countries as a local requirement to introduce any new medical test or device (independently of its regulatory approvals), can significantly delay implementation and does not necessarily provide information of quality. It would be worthwhile to hear more about this in the discussion section, e.g. how important this stage is in the process of a new test implementation and how could it be optimized.

Additional minor comments:

1. On page 3, 1 ^st^ paragraph of the Introduction: “TB was undiagnosed in PLHIV until the time of death in 46% of cases” (ref 4.). It should mention that this was found in resource-limited settings.
2. I suggest proof-reading. For instance, on page 3, 2 ^nd^ paragraph, that, lacks in some clarity as a consequence of the order of phrases. On the other hand, some wording should be reviewed, such as the spelling of *Mycobacterium tuberculosis* in plural as a name in italics, in the same paragraph. Additionally, there is a typo on page 7, 2 ^nd^ paragraph, describing “A systemic review”, instead of “A systematic review”.

I confirm that I have read this submission and believe that I have an appropriate level of expertise to confirm that it is of an acceptable scientific standard.

# Response

**Published:** 2020-04-01

**Authors:** Madhukar Pai

**Affiliations:** McGill University, Canada

**Thank you to the reviewers for your time and detail in evaluating our paper. Please find our comments addressing your comments directly below, in bold.**

This is an interesting study. It aims at reaching better knowledge of the local landscape and barriers for Alere-LAM tuberculosis test implementation in high TB and HIV burden countries.

The study is relevant because the current version of this test has the disadvantage of having a suboptimal sensitivity for TB diagnosis, which can look unattractive to health care organizations from a public health perspective. However, the test can still be very valuable from both patients and clinicians perspectives: it is simple to perform, low-cost, and it can rapidly confirm severe forms of TB in up to 42% of cases with advanced HIV infection, decreasing these patients’ risk of death.

On the whole, I was very well impressed with the paper.

I only have a few minor points to mention:

As one of the study objectives, the authors meant to assess the current landscape of Alere-LAM use in high TB and HIV burden countries. It is striking that 7 out of 11 respondents reported having included the test in their national policies, but not having implemented it yet. I wonder whether or not the authors verified local policies and algorithms for Alere-LAM testing for each of these countries. If not, the discussion should mention if a response bias may have partially accounted for this finding, which could have been particularly relevant when only one respondent participated per country.

**At the time of data collection, the answers reported by questionnaire respondents appeared to be consistent with available data regarding existence of country LAM policies. However, the information that was available only considered “is there a policy, yes/no”, as opposed to implementation of the policy. This discrepancy is what led us to our own questionnaire, with more nuanced findings than just the existence of a LAM policy.**

It is an interesting finding of this study that the need for “evaluation or local pilots studies” was the second most reported barrier for Alere-LAM urine test use. This, which is a common practice in many countries as a local requirement to introduce any new medical test or device (independently of its regulatory approvals), can significantly delay implementation and does not necessarily provide information of quality. It would be worthwhile to hear more about this in the discussion section, e.g. how important this stage is in the process of a new test implementation and how could it be optimized.

**This suggestion has been incorporated into the Discussion (paragraph 4).**

Additional minor comments:

On page 3, 1st paragraph of the Introduction: “TB was undiagnosed in PLHIV until the time of death in 46% of cases” (ref 4.). It should mention that this was found in resource-limited settings.

**This has been noted.**

I suggest proof-reading. For instance, on page 3, 2nd paragraph, that, lacks in some clarity as a consequence of the order of phrases. On the other hand, some wording should be reviewed, such as the spelling of Mycobacterium tuberculosis in plural as a name in italics, in the same paragraph. Additionally, there is a typo on page 7, 2nd paragraph, describing “A systemic review”, instead of “A systematic review”.

**These errors have been corrected.**
